# When open data closes the door: A critical examination of the past, present and the potential future for open data guidelines in journals

**DOI:** 10.1111/bjso.12576

**Published:** 2022-09-08

**Authors:** Annayah M. B. Prosser, Richard J. T. Hamshaw, Johanna Meyer, Ralph Bagnall, Leda Blackwood, Monique Huysamen, Abbie Jordan, Konstantina Vasileiou, Zoe Walter

**Affiliations:** ^1^ Department of Psychology University of Bath Bath UK; ^2^ Department of Social Care and Social Work Manchester Metropolitan University Manchester UK; ^3^ School of Psychology The University of Queensland St Lucia Queensland Australia

**Keywords:** content analysis, ethics, journal guidelines, journals, open data, open science, psychology, qualitative, qualitative methods, quantitative, social psychology, social sciences

## Abstract

Opening data promises to improve research rigour and democratize knowledge production. But it also presents practical, theoretical, and ethical considerations for qualitative researchers in particular. Discussion about open data in qualitative social psychology predates the replication crisis. However, the nuances of this ongoing discussion have not been translated into current journal guidelines on open data. In this article, we summarize ongoing debates about open data from qualitative perspectives, and through a content analysis of 261 journals we establish the state of current journal policies for open data in the domain of social psychology. We critically discuss how current common expectations for open data may not be adequate for establishing qualitative rigour, can introduce ethical challenges, and may place those who wish to use qualitative approaches at a disadvantage in peer review and publication processes. We advise that future open data guidelines should aim to reflect the nuance of arguments surrounding data sharing in qualitative research, and move away from a universal “one‐size‐fits‐all” approach to data sharing. This article outlines the past, present, and the potential future of open data guidelines in social‐psychological journals. We conclude by offering recommendations for how journals might more inclusively consider the use of open data in qualitative methods, whilst recognizing and allowing space for the diverse perspectives, needs, and contexts of all forms of social‐psychological research.

## INTRODUCTION

The move towards open science has been profound in social psychology, where initial evidence for the reproducibility crisis in the social sciences was first reported (Open Science Collaboration, 2015). Many articles published since have argued that open data is a key feature of rigorous, reproducible, and generalizable research, and an important part of a transition towards the improvement of science (Obels et al., 2020). As a consequence, researchers are increasingly encouraged to provide open data, with requirements to provide open data from prominent journals (e.g., Nature and Scientific Data Policy) and funders (e.g., NIH and UKRI), and support for open data from professional societies (e.g., APA and BPS). One action adopted by journals to incentivise open data is offering “badges” on publications where authors have made data publicly available. Promoted by the Center for Open Science, this system is designed to reward and signal open data practices and is, at time of writing, offered by over 75 journals (*Center for Open Science*, 2022). When *Psychological Science* adopted badges in 2014, there was a marked increase in articles reporting data sharing, from 2.5% before to 22.8% in the following year (Kidwell et al., 2016). While open data appears to be increasingly normative in quantitative psychological science, there are significant ongoing debates within qualitative perspectives in social psychology regarding whether, how, and why data should be “opened” (DuBois et al., 2017). These debates precede the recent open science movement in psychology, and raise numerous epistemological, methodological, and ethical opportunities and challenges for qualitative open data.

Many researchers who use quantitative research approaches remain unaware of wider debates about open data within qualitative research. Qualitative research is typically underpinned by different ontological and epistemological philosophies, yet is often held to the same criteria as quantitative research when submitting for publication (Levitt et al., 2018). This is evident in prominent guidelines in the open science movement. For instance, the Transparency and Openness Promotion guidelines (TOP; Nosek et al., 2015, p. 1424) specify three levels of data‐sharing standards in journals. These range from stating “whether data are available and, if so, where to access them” to “data must be posted to a trusted repository, and reported analyses will be reproduced independently before publication”. However, what constitutes “data” is currently not specified in initiatives such as the TOP guidelines and open data policies of prominent journals. Similarly, research examining psychologists' perceptions or practices of “data‐sharing” oftentimes assumes that the data in question is quantitative and fails to address qualitative research explicitly (e.g., Houtkoop et al., 2018; Martone et al., 2018). This lack of acknowledgement or awareness of the debates and nuances inherent in opening qualitative data is concerning in a context where quantitative perspectives dominate and many psychology journals already favour quantitative work for publication (Greenhalgh et al., 2016; Riley et al., 2019). Notably, this may exacerbate biases in what research is published in our journals and compromise the real‐world impact of qualitative research.

This article is presented in three parts. The first provides a primer for the historic debates surrounding open qualitative data, designed for researchers both new to and already familiar with qualitative approaches to psychological research. The second uses a content analysis of open data policies in social psychology journals to assess current journal guidelines for open data in social psychology research. Third, and to conclude, we discuss how journals might better accommodate qualitative research in regard to opening data, including provision of guidelines that promote, rather than detract from, rigorous and thoughtful research practices as well as improvements to training and reviewer assignment. Along with the other articles in this special issue, we hope this article will contribute to a broader discussion within the social psychological community regarding the ways in which we can promote and adopt open‐science practices, whilst still recognizing and allowing space for the diverse perspectives, needs, and contexts of all forms of social‐psychological research.

## PART 1. OPEN QUALITATIVE DATA: HISTORIC DEBATES AND CHALLENGES

### Multiple epistemologies and perspectives on sharing data

Quantitative research typically connects to the epistemology of positivism: the idea that the truth of the world is observable, measurable and can be uncovered through objective phenomena and data points (Coolican, 2018). Qualitative research methods are, by comparison, interconnected with a plethora of research epistemologies and ontologies: ways of understanding knowledge and the world, respectively (Willig, 2013). Qualitative researchers draw on a variety of research methods and approaches, and their research occupies numerous research paradigms (for a comprehensive discussion on the diversity of qualitative research in psychology and paradigms associated with qualitative research see Madill & Gough, 2008). In Table 1, we detail some of the most common *epistemologies* in social psychological research, summarizing their view on what might constitute “data,” as well as providing example studies that adopt these positions.

**TABLE 1:** Brief overview of common epistemological stances in qualitative psychological research

Epistemological stance	View on data	Example study
Realist	Data represents reality. Participant accounts, or observations about participants are taken at face value as truth. There is an objective “reality” that can be explored through words and actions (not just numbers).	A qualitative perspective on multiple health behaviour change: Views of smoking cessation advisors who promote physical activity (Everson‐Hock et al., 2010).
Critical Realist	Reality exists but data is not a direct reflection of it and must be interpreted (e.g., by exploring social meanings) to further our understanding of the perspectives discussed by participants.	Men's perspectives on their grooming practices and appearance concerns: A mixed methods study (Hamshaw & Gavin, 2022).
Phenomenological/Experiential	Data is used to explore the meanings individuals attribute to the world. Participant interpretations and experiences are prioritized. There is more than one reality/truth.	Play hurt, live hurt: Living with and managing osteoarthritis from the perspective of ex‐professional footballers (Turner et al., 2002).
Constructionist	Data allows for the exploration of discursive practices. It offers insights into the construction rather than the description of realities.	“She'll be right”? National identity explanations for poor sexual health statistics in Aotearoa/New Zealand (Braun, 2008).

The varied epistemologies seen in qualitative approaches influence the researcher's perspective on the research question and all aspects of the research process including methodologies, what constitutes research “data,” how the data are managed and analysed, and how findings are communicated. Combined with epistemological position, data collection methods can also have implications for the treatment of qualitative data gathered. In Table 2, we illustrate a number of different qualitative *data collection* approaches and highlight some examples of how published articles treated their data.

**TABLE 2:** Brief overview of data collection methods in qualitative psychological research, with example studies and associated treatment of data

Type of data collection method	Example study	Example study's treatment of data
Collaborative	Preregistering qualitative research: A Delphi study (Haven et al., 2020).	Haven et al. (2020) describe the use of a Delphi procedure to formulate recommendations for preregistering qualitative research. Data was collected online using iterative surveys and feedback reports in‐between surveys. Survey data consisted both of closed and open‐ended responses. Survey data is available on the OSF in .sav (SPSS) proprietary software format. Responses to open‐ended questions within the survey or to feedback reports are not available.
Interview/Focus group	Women survivors of intimate partner violence talk about using e‐health during pregnancy: A focus group study (Fernández López et al., 2022).	Fernández López et al. (2022) used semi‐structured focus groups to explore experiences of survivors of intimate partner violence during pregnancy. Focus group discussions were audio‐recorded and transcribed. The authors note that the datasets are not publicly available to protect participants' privacy but can be made available upon reasonable request.
Naturally occurring/Archival	Young men's body dissatisfaction: A qualitative analysis of anonymous online accounts (Whitaker et al., 2021).	Whitaker et al. (2021) explored young men's body dissatisfaction in online accounts. Here, data are first‐person accounts in a newspaper article and responses to these accounts. The article provides citations and references to datasets utilized, but no means of accessing the news article or responses.
Observational	Women‐only swimming as a space of belonging (Lenneis et al., 2022).	Lenneis et al. (2022) used participant observation in combination with other data collection methods to explore women‐only swimming as a place of belonging. Field notes, including reflexive notes, are captured and excerpts presented in the article.
Structured	Perceptions of climate change imagery: Evoked salience and self‐efficacy in Germany, Switzerland, and Austria (Metag et al., 2016).	Metag et al. (2016) conducted a Q‐sort study to explore the effects of climate change imagery. Data consisted of the sorting decisions and associated interviews. Data availability is not discussed.

*Note*: The data collection categories utilized in column 1 have been adapted from the comprehensive overview by Madill and Gough (2008).

The diversity of data treatment represented in this brief sample shows how differing qualitative epistemologies and data collection methods shape the answers to the questions of what data to share, as well as when, where, and why to share it. Some perspectives might consider open data as aligned with research values and aims, whilst others will regard open data as antithetical to research values and aims (see Mauthner & Parry, 2009). Accounting for this complexity is crucial to engaging researchers who use qualitative methods with open data policies and practices. However, the extent to which journal guidelines take such complexities into consideration, or differentiate between qualitative and quantitative understanding of “data,” has not been systematically examined.

Despite the plurality of qualitative research in psychology, Madill and Gough (2008, p. 255) argue:

Although diverse, we argue that there is utility in maintaining the category of *qualitative research*: The field is often defined in default as “not quantitative”; it has an identifiable history in psychology; and the recent drive to create relevant organizations is based on a sense of shared identifications and professional interests.

In this article, we argue that the imperatives of open science pose a disproportionate disadvantage and introduce challenges for qualitative researchers. We do not assume qualitative research is homogenous and that open science agendas will have similar implications for all qualitative researchers. However, as the quote above suggests, what connects qualitative research approaches is they are *not* quantitative research. Open science approaches and practices have been developed in response to challenges in quantitative research (such as the replicability crisis), and with quantitative research methods and data in mind. What many qualitative researchers have in common is that they face pressures to comply with practices and guidelines associated with open science not designed with our prominent research approaches, methods, and data types in mind. The following sections will discuss challenges that qualitative researchers, using different qualitative methods and researching different topics with different communities, might encounter as demands for opening data rub against research practices, methods, values, or ethics in various ways. We will discuss how some qualitative researchers have approached the questions of why to share data, to what extent, and where data should be shared, and demonstrate the complexity of the open data question for qualitative researchers

### Rigour, replicability, and opening qualitative data

The core reasons to provide open data are commonly stated as improving rigour and facilitating replication, as well as inviting collaboration and allowing others to understand and further build on your work (Borghi & Van Gulick, 2021; Fecher et al., 2015). In quantitative research, rigour is analogous to reproducibility and generalizability, both of which can be assessed when the analysis tools and data are transparent. Research data is thus made open to evidence objectivity and rigour (Alberts et al., 2015). Contrastingly, for some qualitative research in social psychology, rigour is demonstrated by different and more complex standards that cannot be accommodated simply by opening data. Mays and Pope (1995), for example, argue that for some more realist qualitative approaches, such as grounded theory, the objective of evidencing rigour should be “to create an account of method and data which can stand independently so another trained researcher could analyse the same data in the same way and come to essentially the same conclusions; and to produce a plausible and coherent explanation of the phenomenon under scrutiny.” In this example, evidencing rigour is more than making your data openly accessible, it also involves providing other trained researchers with the methodological tools to understand and be convinced of the credibility of your analysis.

Often, opening qualitative datasets such as interview transcripts is not sufficient to establish rigour and the analytic story of the research. Rather, what is needed is evidence to help understand the process of analysis. Johnson and Waterfield (2004) argue measures such as audit trails and reflexivity statements should be used to help other researchers understand how the research was conducted and how it evolved over time. Thus, the concern here is with evidencing thoroughness of analysis through transparency about the decisions and steps made in the analytic process and reflection on those elements of researchers' subjectivity that might have influenced the approach. Congruent with good qualitative teaching and the work of Blignault and Ritchie (2009), the goal of published qualitative research should be to reveal both the “wood and the trees,” that is, a broad understanding of the phenomenon under study as well as relevant idiographic detail concerning a particular element of the broad phenomenon. From this perspective, the problem qualitative researchers face ‐ particularly in journals geared to quantitative methods ‐ are constraints on their ability to explain their decision‐making through small word counts or prescribed subsections favouring parsimony over depth and complexity.

Adding further complexity, qualitative researchers who work within a social constructionist paradigm welcome plurality of meaning and treat their findings as just one of many possible readings of the data (Hollway & Jefferson, 2000; Huysamen & Sanders, 2021). From a social constructionist perspective, language should be understood within the specific socio‐historical context in which it is produced. Similarly, researchers' readings and interpretations of the data must be situated in time and place; these are shaped by their personal, political, and theoretical positionings, and will shift and change over time (Huysamen, 2022). Thus, replicability is typically not a criterion for measuring research rigour and quality in social constructionist approaches. Indeed, for social constructionist approaches, the very notion of wanting to replicate findings, let alone believing opening data would allow for this, is erroneous. This is not to say constructionist approaches might not value open data; from the perspective of recognizing multiple realities and truths, the opportunity to generate and make available secondary data might be valued for making possible new analyses and interpretations from different perspectives and positionalities.

### Reflexive practice and opening qualitative data

Some realist approaches to qualitative research, where analysis is mostly descriptive and recounts what participants say verbatim, may consider anonymized full transcripts sufficient evidence for a rigorous analysis of the data. Readers may be expected to be able to understand the analysis from reading the transcripts alone. However, other approaches in social psychology, such as Hollway and Jefferson's (2000) *Psychosocial Approach* attend closely to the researcher's positionality, interview context, and impact of the interviewer‐participant dynamics on the data collected and the knowledge subsequently produced. In such approaches, a reflexive journal or notes may be considered an important tool for assisting the interpretation and analysis of the data. In approaches such as Huysamen's (2022) *Critical Reflexive* approach, the researcher systematically keeps reflexive accounts throughout the research process which are considered *part* of the research data. But if reflexive accounts are crucial to understanding research data or even to be research data, what are the implications for opening such data? While these researchers will argue that reflecting on questions such as their own positionality and power are essential to understand the research in context, in approaches where researchers are encouraged to reflect on their own biases, anxieties, prejudices, hopes, and expectations, making reflexive accounts open may not be appropriate and may have far‐reaching implications (Huysamen, 2022). When considering the question of open qualitative data, it is crucial to ask what data should be open, and why? What data is enough to convince readers of the rigour of qualitative work? And what are the implications of other researchers accessing and using different kinds of open qualitative data?

### Ethical and political considerations of opening qualitative data

In addition to the epistemological and methodological complexities described above, there are several ethical and political considerations which should be discussed in relation to opening qualitative data. For example, when conducting research involving illegal, sensitive, or stigmatized topics, maintaining the anonymity of participants is likely to be a key ethical responsibility for researchers in order to protect participants from physical, emotional, and reputational harm (Huysamen & Sanders, 2021). Researchers in social psychology submitting their studies for ethical review are likely to find that research ethics committees expect evidence of a clear data management plan, which usually includes ensuring participants' anonymity is maintained. While quantitative datasets can often be anonymized with little effort, the burden of anonymizing qualitative data often demands more time and resources, and decisions about what should be anonymized are not straightforward. For instance, when conducting research with small minority groups, participants might be easily identified by members of that group (or interested external authorities) simply by their association with the topic, community, or practice. In these instances, the person anonymizing the data might not be familiar enough with the community or group to know which minor details present in a transcript may reveal a participant's identity. Where there is an expectation that data should be anonymized before opening the data, this burden is likely to be greater for those with qualitative datasets, requiring more researcher time and thus more funding to effectively address.

Furthermore, there is no guarantee that qualitative transcripts that are considered fully anonymized at publication will remain unidentifiable. Computational authorship attribution is an active field of research that provides tools facilitating the identification of the speaker or writer of a text (Barlas & Stamatatos, 2020; Stamatatos, 2009), and it would be sensible to expect the continued development of such technologies.

What may also change over time is the political and legal context; research not considered legally or ethically sensitive at the time of publication may become sensitive and challenging following publication. For instance, the UK government's proposed policing bill, if enacted, would criminalize protesters who cause “serious annoyance,” as well as Gypsy, Roma, and Traveller groups who pitch on private land (Casciani, 2021). Abortion law in the United States is another example of how fast the political and legal context might change. When we wrote the first submission of this article, abortion was legal in the United States. When we submitted the final revisions, the *Roe vs. Wade* ruling in the US protecting abortion access had been overturned by the Supreme Court, making abortion illegal or inaccessible in many US states (Sun, 2022). The dramatic change to the legal status of abortion access in the US, within even one article's publication timeline, reveals the complexity of openly publishing data on an issue that can then become highly sensitive and potentially incriminating for participants. Thus, researchers, reviewers, and editors should be mindful of the complex and changing social dynamics surrounding their research topics in order to mitigate any severe consequences opening data might have for participants.

In contrast, carefully negotiating “legitimate sensitivities” in secondary analysis of open data can be a way to increase the study of sensitive topics, adding value to communities researched, and reduce shame around important social issues. Branney et al. (2019) suggest that a context‐consent meta‐framework should be employed for secondary qualitative studies using open data—particularly on sensitive topics—where researchers interrogate the context of the original research and its ethical, practical, and theoretical suitability for their secondary research questions. Providing a comprehensive description of the research design, ethics, context, and process alongside any open data can thus enhance the ethics and rigour of secondary opened data analysis.

From a commercial and legal perspective, data which is deemed to be owned by private entities (e.g., companies, NGOs, corporations) may also not be openly accessible due to copyright, intellectual property, and concerns over competition (Zuiderwijk et al., 2016). Research concerning, and conducted in collaboration with, commercial organizations is becoming increasingly important for social psychologists seeking impact in their research, and a requirement for open data may limit the ability of applied researchers to publish “protected” works. A company may be less willing to work with a researcher they know will open the data, compared with an independent consultant working outside of academia who faces no such requirement. Thus, considering the complex legal, political, commercial, and social conditions of our research in the present day and the future is vital for wading through the ethical quagmires some forms of data sharing might present in the long‐term.

One cited benefit of open data is to provide datasets for use by other researchers for secondary analyses, thus accelerating advancement of psychological knowledge (Gewin, 2016). However, for qualitative data where the context and the researcher–participant relationship in and of itself is a core component of the research, this may not be ethically or empirically responsible. In‐depth qualitative methods such as ethnographic methods, Participatory Action Research, and unstructured interviews often involve an intensive trust‐building process between researchers and the individuals and communities they research (see Ellis, 1995). Researchers adopting these methods may collect broad and rich accounts from participants, detailing intimate aspects of their lives, which may or may not be directly relevant to the research question(s). These participants' accounts are produced within a particular context, and it is the researcher's ethical responsibility to represent participants' accounts in ways that reflect these contextual specificities within which it was produced, and in keeping with the ways participants intended for them to be represented and used. This is in line with ethical critiques about the coloniality of so‐called “helicopter research” that extracts research data from communities without making efforts to give back (Montour & Macaulay, 1988). Where data is openly available, researchers who have never had contact with a community can extract their perspectives, out of context, for their own gain without seeking consent from the original participants to reuse the data for different research purposes to those which participants had intended (Couldry & Mejias, 2019). Thus, transforming every narrative shared by our participants into public data for reuse is neither automatically best practice nor does it in every instance represent the most ethical approach to data management (Tuck & Yang, 2013).

Feminist decolonial researchers (e.g., Ahmed, 2013; Simpson, 2007; Tuck & Yang, 2013) have long championed an approach to research ethics and accountability that stands in stark contrast to the blanket approach of the open science agenda. Rather than the imperative to automatically make public all of participants' qualitative accounts, they speak to the ethical responsibility of knowing when to hold back. This recognizes the long history of data from research conducted with marginalized groups (such as indigenous people and autistic people) being used to their detriment or weaponized (Walter & Andersen, 2016). Tuck and Yang (2013) suggest “we come across stories, vignettes, moments, turns of phrase, pauses, that would humiliate participants to share, or are too sensationalist to publish” (p. 234). Ahmed argues that silence is a strategic response to oppression and “sometimes we might stay silent about some of the findings of our research because we do not trust how those findings might be used by other actors” (Ahmed, 2013:xvi). When responding to pressures to make our data open without discretion, we must attend to questions of power and of who benefits from this and why (Simpson, 2007). Thus, it could be argued that the imperatives of open science may at times directly oppose and prevent researchers from being able to act in accordance with their research ethics of accountability and treat their participants' data with consideration and care.

Yet, as Bishop (2009) argues, no single ethical claim surrounding data sharing is irrefutable. Just as some might argue opening qualitative data may present issues for participant safety, others might argue it is the responsibility of the researcher to ensure participant voices have as much impact as possible on their community through sharing and reuse (a view also supported by Kuula, 2011). In some cases, complete confidentially may not be a participant preference, and thus researchers may wish to explore freedom of choice in such matters (Kaiser, 2009). Some research has suggested that even participants who may be deemed as “vulnerable” may wish to be named in published findings (Grinyer, 2004). As such, Kuula (2011) argues the ethical debates surrounding data sharing should be deepened, and above all, participants must be able to fully consent to any data sharing that may occur before they participate. Therefore, gaining informed consent to share data requires a respectful and reciprocal relationship with participants as well as a careful case‐by‐case consideration of impact. It is insufficient to simply “add an extra box” to our consent forms, or assume consent in hindsight when it was not originally given for this express purpose, as this may not be compliant with current regulation. Rather, gaining informed consent should involve a discussion on the ethical and legal implications of data sharing in the long term, and should ensure participants are helped to grapple with these issues in collaboration with researchers. This further begs the question, are the “open data” made available via journal publication platforms really “open” to participants and their communities to use to further their own agendas and goals? If there is an imperative to open data, arguably there should also be imperatives within social psychology that datasets, and the (oftentimes paywalled) publications they are used in, to be made genuinely free and open, in that they are made available in a location and in a format that may be genuinely accessible and useful to communities, self‐advocacy groups, and grassroot movements. Thus, open data is but a small piece of a larger puzzle questioning how we can increase accessibility, availability, and usefulness of our research to those who may benefit from it.

## PART 2: INTERROGATING CURRENT OPEN DATA JOURNAL GUIDELINES

The epistemological, methodological, and ethical questions surrounding open data discussed so far have been widely explored in the qualitative research literature, and positions vary according to researchers' specific allegiances. However, as quantitative research in social psychology, and funded research in general, moves towards an “open data as standard” model, qualitative researchers are increasingly being asked in journal guidelines to open their data upon publication without due acknowledgement of these complex issues and debates. This is challenging as publication is an important means for researchers to gain research impact, academic esteem, job security, and to publish work which opens up possibilities for future research, collaboration, and real‐world impact. Journals function to a great extent as gatekeepers, filtering who has access to these opportunities and who does not, and journal guidelines to authors impact research design and evaluation. This is particularly the case for lone or early career researchers (ECRs) who may not have mentors to guide them through the informal norms of journal submission (Nicholas et al., 2017). In this way, unilateral or vague open data requirements could function as a blunt instrument that may serve to close the door to publication for many researchers using qualitative approaches or to pressure researchers into practices they believe are “best‐practice” without critically reflecting on the complexities of issues outlined above. To examine the state of the current policies on open data, we reviewed 261 journals in social psychology, exploring their author guidelines for open data requirements and their attention to qualitative data. In doing this, we consider the current adequacy of journal policies, and we offer recommendations for improving the approach of open data guidelines towards methodological, ethical, and epistemological issues moving forward.

## METHODS

### Data overview

A key aim of this study was to investigate the content and availability of open data guidance across social psychological journals, with a specific focus on qualitative research data considerations. To consider journals publishing work relating to social psychology, a Scopus catalogue for all journals tagged with the subject area of social psychology was utilized. This list initially comprised 335 journals, but given the method‐specific nature of this investigation, review journals, journals accepting commissions or internal contributions only, and book series were excluded (*n* = 20), as were journals requiring manuscripts to be in a language other than English or did not provide English language author guidelines due to translation limitations (*n* = 19). After excluding out of print, renamed, merged or inaccessible journals (*n* = 35), this resulted in a final dataset of 261 journals, published by 75 different publishers. An overview of these journals can be found via the Open Science Framework (OSF) at https://osf.io/zhrpn/.

### Analytic approach

Between August and September 2021, journal landing pages, aims and scope overviews, and manuscript/author guidance pages were scrutinized to consider several aspects of open science practice. Coding categories were developed and refined through several conversations about qualitative open data between the author team. Journals were coded using author‐derived forced response options (e.g., yes; no; unclear) in terms of: their acceptance of qualitative work, presence of open science guidance, guidance specific to qualitative submissions, as well as other elements of associated data such as expectations for open data (e.g., requirements for data sharing statements). Additional annotations were also made as part of the coding process, for example, noting any nuance in considerations of open data (e.g., an awareness that not all articles can ethically be open) and use of external guidance materials. Four researchers on the author team carried out the coding, with 20% of the dataset cross‐coded by any two of the four coders before individual coding. Intercoder agreement ranged from *𝜅* = .59–.76 between coding dyads, indicating moderate to substantial agreement between coders (Burla et al., 2008). The full intercoder results and coded dataset are available at https://osf.io/zhrpn. Echoing the arguments presented in the earlier sections of this article, we are unable to openly share the full text of the journal pages we scrutinized due to copyright issues. However, we have provided links and the name of each journal analysed, and we encourage interested readers to visit those links to access the journal guidelines. Discrepancies in initial code assignments were noted, discussed, and resolved through team discussion to ensure consistency before allocating the remaining journals equally across the team. Focusing on reflexivity, each coder also provided a qualitative reflection on the coding process (anonymized reflections are openly available on the OSF page: https://osf.io/zhrpn).

### Positionality statement

Throughout the development of this article, we have been aware of our own experiences and expertize in handling the complex issue of opening data. This work has been produced by a team of researchers in varying career stages, with differing methodological orientations (spanning qualitative, quantitative, and mixed methods), and from numerous fields of psychology (e.g., social, health, and forensic). As a result, we feel our conclusions represent a broad array of perspectives in approaching open data, and those wishing to use qualitative methods. Content analysis coders were all ECRs, which allowed for examination of how people with less publishing experience interpret journal requirements. Coders were a mixture of PhD students and staff members. All coders have experience of teaching qualitative and quantitative methods in psychology at undergraduate level, and experience conducting qualitative, quantitative, and mixed‐methods research. Experience with publishing research among coders ranged from zero to nine publications at time of coding. Overall, this article was a truly collaborative process. Much of our interest in this topic stemmed from the engaging discussions between all authors regarding findings and experiences, some with many years of experience and holding more senior academic posts than others. We were surprised at the commonality of our experiences regardless of career stage, and we identify the issue of qualitative open data as an emerging problem with unclear resolutions relevant to researchers of all experience levels.

## RESULTS

The coded dataset and statistical code used to generate these results is openly available on the OSF at https://osf.io/zhrpn/. Of the 261 journals selected for review, four journals explicitly rejected qualitative work and thus were excluded from further analysis. Of the remaining 257, some journals explicitly listed qualitative work in their remit (*n* = 74), but the majority of journals did not explicitly mention whether they did or did not accept qualitative work (*n* = 183). To explore similarities and differences between journals explicitly welcoming qualitative research compared with journals not mentioning a stance on qualitative research, we split our initial analyses and findings according to their stance on qualitative research (excluding journals explicitly not accepting qualitative work; see Table 3).

**TABLE 3:** Relative frequencies of journal guidelines relevant to open data for social psychology journals by stance on qualitative research

Policy detail	Stance on qualitative research		All relevant journals (*N* = 257)
	Explicitly accepting (*N* = 74)	Unclear (*N* = 183)	
Of *n* (%) journals with open data guidelines …	44 (59.5%)	102 (55.7%)	146 (56.8%)
… has open qualitative data guidelines	2 (4.5%)	1 (0.98%)	3 (2.1%)
… makes use of external policy	31 (70.5%)	76 (74.5%)	107 (73.3%)
… open data “encouraged”	37 (84.1%)	76 (74.5%)	113 (77.4%)
… open data “required”	1 (2.3%)	14 (13.7%)	15 (10.3%)
… open data requirement unspecified	6 (13.6%)	12 (0.98%)	18 (12.3%)
… non‐sharing justification expected	17 (38.6%)	46 (45.1%)	63 (43.2%)
… data availability statement expected	33 (75.0%)	81 (79.4%)	114 (78.1%)

*Note*: “Total relevant journals” represents both journals explicitly accepting qualitative research and those that are unclear about it. Four journals explicitly stated not accepting qualitative research and are not included here.

Table 3 presents findings from the analysis of journals' open data guidelines. Author guidelines on open data were provided in just over half of all journals reviewed (56.8%). This pattern was evident both for journals explicit and unclear about accepting qualitative research. These findings point to a consistent use of open data guidelines by social psychology journals regardless of their explicit endorsement of qualitative research.

Next, we investigated guidelines specific to open *qualitative* data. Guidelines explicitly addressing open qualitative data were very rare, provided by just three journals (2.1% of all relevant journals). All other journals that made reference to “open data” did so without making a distinction between qualitative and quantitative data. Instead, “data” were referred to in a vague way typically centred on quantitative (numeric) data. Of the three journals with some guidelines for qualitative open data, these guidelines provided little specific advice, and often linked to a published article or further ambiguous guideline. Journals were also unclear about *what parts* of the data should be made openly available. We initially coded for journals' specification of what they considered appropriate data to make available, with a view to discussing distinctions of open data guidelines encompassing full transcripts, codebooks, analysis, or other aspects of the qualitative analytic process. Yet during the coding process, it became clear any distinctions made by author guidelines referred to data used in quantitative analyses. If any nuance regarding data sharing expectations was present, this was typically restricted to the acknowledgement that ethical, privacy, legal, or security reasons for not sharing data may exist, without any further elaboration on what these reasons are or guidance as to how they might be addressed by researchers.

To examine journals' expectations for research submissions, we coded for whether open data was explicitly “required” or “encouraged” in guidelines. Open data was encouraged in over three quarters of journals (77.4%), both within those explicitly accepting qualitative research (84.1%) and those unclear about it (74.5%) (Table 3). More than three quarters of journals required authors to provide a data availability statement (a statement detailing if data is openly accessible, and if not, why not) upon submission (78.1%), and more than one third of journals explicitly asked authors to justify their decision to *not* share their data (43.2%). These requirements tended to be slightly more common in journals with an unclear stance on qualitative research (see Table 3).

Most journals with open data guidelines drew upon specific open data policies external to the journal itself (e.g., from the journals' publisher, see Table 4). We also examined the external open data policies cited by journals and provide a summary of those most frequently used. We identified 24 different policies or policy sub‐types; this number should, however, be treated with caution as journal guidelines differ in the way they treat broader guidelines such as the Transparency and Openness Promotion (TOP) guidelines as a single guideline versus detailing their stance on different transparency standards.

**TABLE 4:** Summary of most frequent external open data policies from journals that provide open data guidelines

External Open Data Policy	Used by *n* journals (% of 107 journals with external guidelines)	Summary of policy
Taylor & Francis: Basic Data Sharing Policy	36 (33.6%)	Authors are encouraged to share or make data and materials available (where ethically appropriate to do so), using a recognized data depository. Authors are encouraged to provide a data availability statement, stating where data or materials can be found (and explaining why any data was decided to not be made available).
Transparency and Openness Promotion (TOP) Guidelines	18 (16.8%)	Eight transparency standards: Citation; Data transparency; Analytic methods (code transparency; Research materials transparency; Design and analysis transparency; Preregistration of studies; Preregistration of analysis plans; Replication). Standards can be “adopted singly or collectively,” across three levels of increasing transparency and openness.
Springer Nature: Research Data Policy Type 1	10 (9.3%)	Data sharing and data citation is encouraged. Details of data sharing via repositories are referred to in journals' guide to authors. The journal style guide permits authors to cite publicly available datasets in manuscript reference lists.
Taylor & Francis: Share Upon Reasonable Request Data Sharing Policy	7 (6.5%)	Authors agree to respond to data sharing requests and make data and materials available upon reasonable request (the author should judge if a request is reasonable, and if it is ethically appropriate for the data/materials to be shared). Authors are recommended to deposit data in a recognized data depository prior to submission. Authors are required to provide a data availability statement, stating where data or materials can be found (and explaining why any data was decided not to be made open).
Wiley: Expects Data Sharing Policy	7 (6.5%)	The journal expects that data supporting the results in the article will be archived in a public data repository (where ethically and/or legally appropriate to do so). Authors are required to provide a data availability statement, stating availability or the absence of data, and a link to data repository (if data is made available). Where possible, “scripts and other artefacts used to generate the analyses presented in the article should also be publicly archived.”

Taylor & Francis' Basic Data Sharing Policy was the most common, cited within 14% of the 257 journals explicit or unclear in the acceptance of qualitative research, and within 33.6% of the 107 journals which provided guidelines on open data. In four out of five of the policies in Table 4, these form one of multiple policy‐specific “levels” of transparency. Policies included in Table 4 are broadly at the less strict end of the transparency spectrum. For example, Taylor & Francis' open data policies range from the “Basic Data Sharing Policy” (Table 4) through to the “Open and FAIR” policy, which *requires* authors to make data or materials supporting the results and analyses freely available (with data made available meeting Force 11 FAIR standards). In Table 4, we also included the 18 journals which cited The Transparency and Openness Promotion Guidelines as their open data policy. Six (33.3%) of these journals specified the level (1–3) of the TOP Guidelines, and the remainder referenced components of TOP guidelines without specifying which level these equated to.

We observed that external policies, such as the Taylor & Francis Basic Data Sharing Policy, appeared to have little specificity and relevance to qualitative research and data. This is exemplified in the guidelines of one of the two journals bearing the word “qualitative” in their title: *Qualitative Research in Sport, Exercise and Health*, published by Taylor & Francis. This journal shared their open data policy (Basic Data Sharing Policy, see Table 4) word‐for‐word with that of non‐qualitative journals. This indicates that, even in explicitly qualitative journals, external open data policies are used that do not distinguish between the differing types of data used within qualitative and quantitative research.

### Coder reflections

Detailed reflections from coders can be read at https://osf.io/zhrpn/ as part of our supplementary open data. We briefly summarize our reflections here.

Coders felt guidance was often difficult to find, and obtuse in nature, as one notes: “Having to move between different sections of journals' instructions for authors, their editorial policies, external websites, etc. made it difficult to understand what journals expect of authors.” The irony of open data providing a means for research transparency, but the challenge in locating or understanding guidance associated with this was not lost on us. Furthermore, the lack of detail in what might be suitable to share or why approaches may vary, left us feeling perplexed in terms of how open data approaches may work with our future research. As one coder noted: “open science, to me, often felt like lip service for many journals, or guidance was just added in without consideration of various methods.” Many journal guidelines requiring open data did not provide enough detail for the rationale behind open data to effectively guide researchers through expectations of the process, for instance: “it was difficult to distinguish between when open data was specified as ‘encouraged’, ‘required’, and ‘expected.’” Seemingly simple changes in guideline wording can be difficult for researchers to interpret, and may have large downstream consequences for how an article is considered following submission.

The British Psychological Society (2020) guidelines state that open data should be “as open as possible, and as closed as necessary” (p. 1). However, one coder commented on how this vagueness might work against qualitative researchers in the publication process: “The ‘[share] what you can’ bar that is relatively stable for standardized data becomes reliant on authors and reviewers agreeing on ethical and methodological issues in highly idiosyncratic contexts.” They argue this flexibility is especially problematic for ECRs: “As an ECR, that very easily translates to being lost in unwritten rules. Should we have a stab in the dark at whether BPS position believes our reason for not opening data qualifies as ‘ethical grounds’? Or should we make it open just in case?” For all coders, journal guidelines did not serve to alleviate any confusion in this regard.

Coders were also concerned with an absence of guidance towards open data in explicitly qualitative journals: “… more qualitative‐orientated journals skipped over open data.” Given the move in social psychology towards open data as standard, the observation that some journals made no note of open data could mean qualitative researchers get “left behind,” and their published work becomes more open to critique. This is concerning, the coder reflects, given “many of the principles of open science are already being demonstrated by qualitative researchers. But this practice may be less obvious than an open science badge above a title, or an entire quantitative dataset being available in a data repository.”

Overall, coders ended the analysis surprised and concerned by the lack of clarity in open data approaches both generally and in relation to qualitative research specifically. One coder even reflected that “I don't have any better understanding of what qualitative data journals wish to be made available than I did before starting this project.” If journal guidelines are currently difficult to interpret even for those immersed in a project about open data, we wondered how those who are trying to submit a single article to a journal might struggle even more.

## PART 3: GENERAL DISCUSSION AND RECOMMENDATIONS FOR A MORE INCLUSIVE FUTURE FOR OPEN QUALITATIVE DATA

When we set out to write this article, it was with what may have been a naive expectation that within the social psychology journals analysed there would be a clear vision for open science and open data that would provide guidance to researchers on opening quantitative data for publication. We assumed this would form the basis of our discussion on the applicability and implications of this guidance for publishing qualitative research. Instead, our analysis shows that whilst what open science and open data might look like for qualitative research is particularly opaque, the message is not much clearer for quantitative research. While the principle of open data was prevalent among many journals, guidelines were often generic and non‐specific to qualitative data (even in journals with an explicitly qualitative focus). A broader point that became clear when examining external guidelines was the open science movement itself has demonstrated little engagement with qualitative research. The same pattern of open data being “encouraged” in explicitly qualitative journals is mirrored in those journals not accepting qualitative research, with three out of four explicitly non‐qualitative journals encouraging but not explicitly *requiring* data sharing. To what extent this encouragement for open data is heeded in practice by authors and reviewers of different journals will need to be the focus of follow‐up analyses and will undoubtedly be of interest to those trying to navigate the maze of publication with little more than a map copied and pasted from a neighbouring maze.

There appear to be two approaches many journals take in offering open data guidelines. The first is to refer to external guidelines or exemplars, many of which are equally opaque in their definition of data and reference to qualitative work. The second is to invite authors to make, and in some cases provide justification, for their own judgements. Regarding the former, open data requirements were often used as a “blunt instrument,” without due regard for the epistemological, ethical, and practical nuances of data. Further, definitions of what constitutes data (e.g., raw data, transcripts, codes, and reflections) and what form data‐sharing should take, were not included in journal guidelines. This was even the case for some journals which explicitly targeted qualitative research. In regard to the latter, what was typically referenced were “ethical” considerations (i.e., not methodological or epistemological), often relating to respect for privacy and confidentiality of participants, or “sensitive” data, but again providing no guidelines for what this looks like in practice or ways to ensure data is anonymized. This suggests a reluctance thus far on the part of journals (and one assumes editors) to engage with the kinds of debates we opened with, some of which will have a bearing on some quantitative research. Active engagement and leadership in these debates is particularly critical in a context where current calls for decolonization are presenting new and potentially conflicting challenges (e.g., requiring consideration of power in the use and misuse of data).

Some may argue opaqueness is the qualitative researcher's friend; who could argue with the fairness of leaving it up to the researcher to decide what is appropriate in their circumstance? However, this fails to account for power and the day‐to‐day exigencies of authoring, reviewing, and editing. In a context where (a) bias exists towards qualitative research and (b) qualitative researchers are aware of and may orient to that bias, there are certain perils. Research may be summarily excluded because the author violates the editors' (or reviewers') personal values about open data; or authors (particularly those with low power) may compromise their own values and those of their peers by sharing data that may potentially harm participants, communities, and the profession. If approaches to open data regarding qualitative research are to continue in the current vein, there is an additional concern researchers may even be discouraged from conducting the kind of research qualitative approaches have been crucially exploring. As such, open science needs to be sensitive to the research, rather than the research simply adapting to open science.

The open science movement has many laudable aims which align with the values of qualitative researchers. These include greater transparency and integrity in our research and the democratization of knowledge use and production. In terms of transparency and integrity, we agree with many who have gone before us that what is most important is (a) for qualitative research to be given the space needed in the pages of our journals to explain the research approach and findings, and (b) for far more attention to be given to training in qualitative methods in our institutions (and what is covered in this training to be reflected in what is published). Indeed, until our journals have editors and reviewers with sufficient knowledge and regard for the range of epistemological, methodological, and ethical considerations in making qualitative data “open,” this expectation (even when qualified as a choice) remains problematic.

The arguments around the democratization of knowledge are more complex, particularly when we consider who the imagined users of our shared data are and how data might be used (or misused). For some qualitative researchers, particularly those working with vulnerable or marginalized communities, the idea of sharing data with anyone other than participants is anathema to their professional values and is seen as compromising the future of such research. More explicit recognition and articulation of such concerns in journal guidelines would shift the onus to the warranting of sharing (as well as not sharing). Moreover, even where dangers in sharing are not immediately evident, there are strong arguments for a cautious approach to making qualitative data open. Journals (and publishers) need to have a view on the ethical and legal contingencies of sharing data (e.g., what consent is required and how ethical standards apply for those using data) and cannot simply leave it to authors.

Following from the discussion above and findings of our journal guideline analyses, we present the following recommendations:

First, journal guidelines, and the open science movement in general, need to recognize the breadth and complexity in qualitative approaches. We are in accord with the BPS Open Data Position Statement which takes a “principles”‐based approach of “as open as possible; as closed as necessary” and cautions against being prescriptive. We do, however, argue that additional guidance from journals and professional societies is needed. Reflecting this more “principles”‐based approach, journal guidelines could include statements on, or examples of, what *might* constitute transparency, openness, and rigour for different qualitative approaches. This would need to be done in a way that signals recognition of what opening data means for different data types and for different epistemological, methodological, and ethical positions. For instance, it would be important to communicate recognition and support for open science practices already prevalent in some qualitative research approaches such as providing audit trails, reflexivity statements, and codebooks through to involving participants in all stages of the research process. Equally, it would be important to alert reviewers and authors to the kinds of considerations that might affect decisions not to open data (such as those discussed in part 1 of this article, e.g., considerations of who “owns” data, and perspectives on anonymity and truth). Regarding the assessment of open data, we believe reviewers and editors should assess articles on the clarity and quality of their particular argument for *or* against opening data, rather than solely on whether the data has been made openly available. Such an approach would allow for greater flexibility and would enable researchers to evidence rigour in their research even if they are unable to open their data.

Our findings highlight the value of developing clear author guidelines for the treatment of open data that reflect and respect the heterogeneity of research practices, principles, and epistemologies used within social psychology. We would encourage journals to consider a more inclusive approach to open data by co‐producing any guidelines or recommendations on open data in collaboration with a diverse range of their readership, editorial team, and contributors, representing many theoretical and methodological orientations (both qualitative and quantitative). A co‐produced approach to the assessment of open data would allow for the full and fair consideration of the myriad challenges researchers, both qualitative and quantitative, might face when opening their data. Any guidelines or actions resulting from this process should be reviewed and updated regularly to ensure a consistent engagement with emerging social issues and technologies relevant to open data and each journal's research context. We hope that following this prescient special issue, the BJSP may lead the way for social psychology by developing the first set of truly co‐produced journal position statements and author/reviewer guidelines on open data, giving particular voice to the challenges qualitative researchers may face as featured in this article.

Second, word limits and narrow aims and scopes are challenges that all researchers, but especially qualitative researchers, face when searching for a location for their work. One way for journals to help evidence the rigour of research in general may be not to force open data as standard, but rather to reimagine scientific communication and allow significantly more flexibility in word counts and supplementary materials so that all authors can give a comprehensive explanation of their research. Additionally, rather than expecting a crude and ill‐defined description of the availability of the data, journals could instead guide authors towards including a more detailed account of decision‐making regarding open data alongside broader decisions about epistemology, methods, and ethics in the manuscript itself. In providing more guidance, journals would need to be more explicit about whether they welcome qualitative research at all. This step in itself would mean a significant improvement for qualitative researchers when considering what is expected of them as they submit work for publication.

Third, more generally within social psychology, there is a need to provide further accessible training and resources around transparent research practices that include the complexities of issues surrounding open data. It is worth noting such training and resources would benefit researchers not only utilizing qualitative methods but also quantitative approaches. For example, debate still exists within quantitative research regarding how to determine whether a dataset is sufficiently de‐identified. Additionally, current debates around decolonization are challenging all researchers to consider issues of power around who produces research, who has access to data, and how data is used. We need to equip researchers with the skills to ensure participants are treated with dignity and respect, particularly when considering inequalities in power and issues of consent. Moreover, given the opaqueness of data sharing guidance, researchers cannot place the burden on participants to consent to their data being made open if we, as researchers, cannot articulate what this will entail.

Finally, editors and reviewers come from within academic ranks and both their motivation and ability to understand and evaluate how data might be treated across different qualitative approaches reflects systemic issues within our discipline. Equipping editors and reviewers for this task would require providing them with the kind of training absent from many of our research methods programs and would likely be considered well beyond the scope of journals. We do, however, recommend journals provide practical guidance on open data to editors and reviewers. This could include information of epistemological approaches, the ethical and legal issues related to opening the data (e.g., can the data be anonymized, was consent obtained for data sharing, how great is the potential for harm, is this a vulnerable population?) and clarity on what could constitute data in qualitative research. For example, data (as a term) was all too often used to refer to quantitative datasets; guidance would do well to acknowledge the variety of data available within a qualitative project (e.g., interview transcripts, field notes, reflective journals). Ideally, journals could also commit to ensuring articles are edited and reviewed by at least one person who has relevant methodological expertise and only they are asked to comment on methods and related matters.

## CONCLUSION

Our analysis adds to the multitude of voices in qualitative social psychology considering the many practical, ethical, and epistemological issues researchers face in opening their data. We demonstrate that journal guidelines for open data, at present, do not reflect the diversity and complexity of psychological research. We believe journals must do more than lip service to open science in their guidelines and should actively help authors to consider and address the many potential challenges implicated in opening their data. While they may be well‐intended, we demonstrate that poorly articulated guidelines on open data may in fact detract from rigour and transparency, and instead introduce challenges that researchers at all career levels may find difficult to navigate alone. Open data policies may still be an “open door” to further research, rigour, and social impact. However, as we demonstrate, a one‐size‐fits‐all approach to opening data can easily become a barrier for rigorous and considered research, and we encourage journal editors to consider how opaque guidelines may close the door for certain research methods or approaches.

## AUTHOR CONTRIBUTIONS

**Annayah M. B. Prosser:** Conceptualization; data curation; investigation; methodology; project administration; supervision; writing – original draft; writing – review and editing. **Richard J. T. Hamshaw:** Conceptualization; data curation; investigation; methodology; project administration; writing – original draft; writing – review and editing. **Johanna Meyer:** Data curation; formal analysis; investigation; methodology; validation; writing – original draft; writing – review and editing. **Ralph Bagnall:** Formal analysis; investigation; methodology; writing – original draft. **Leda Blackwood:** Writing – original draft; writing – review and editing. **Monique Huysamen:** Methodology; writing – original draft; writing – review and editing. **Abbie Jordan:** Writing – review and editing. **Konstantina Vasileiou:** Writing – review and editing. **Zoe Walter:** Writing – review and editing.

## CONFLICT OF INTEREST

All authors declare no conflict of interest.

### OPEN RESEARCH BADGES

This article has earned Open Data and Open Materials badges. Data and materials are available at https://osf.io/zhrpn.

## Data Availability

The coded data examining journal guidelines used in the content analysis of this article, as well as anonymized qualitative reflections from coders, are publicly available on the Open Science Framework at https://osf.io/zhrpn. Illustrating one difficulty in sharing qualitative data, the original journal webpage content we coded for our content analysis during August and September 2021 is under third‐party copyright and cannot be freely redistributed or stably linked. Interested readers are invited to use the tools provided in this article to access the original materials summarized in our coded dataset.
